# Isolated terawatt attosecond hard X-ray pulse generated from single current spike

**DOI:** 10.1038/s41598-018-25778-x

**Published:** 2018-05-10

**Authors:** Chi Hyun Shim, Yong Woon Parc, Sandeep Kumar, In Soo Ko, Dong Eon Kim

**Affiliations:** 1 Department of Physics, Pohang University of Science and Technology, Pohang, 37673 Korea; 2 Pohang Accelerator Laboratory, Pohang University of Science and Technology, Pohang, 37673 Korea; 3 Department of Physics, Ulsan National Institute of Science and Technology, Ulsan, 44919 Korea; 4 Department of Physics, Center for Attosecond Science and Technology, Pohang University of Science and Technology, Pohang, 37673 Korea; 5 Max Planck POSTECH/Korea Res. Init., Pohang, 37673 Korea

**Keywords:** Free-electron lasers, X-rays, X-rays

## Abstract

Isolated terawatt (TW) attosecond (as) hard X-ray pulse is greatly desired for four-dimensional investigations of natural phenomena with picometer spatial and attosecond temporal resolutions. Since the demand for such sources is continuously increasing, the possibility of generating such pulse by a single current spike without the use of optical or electron delay units in an undulator line is addressed. The conditions of a current spike (width and height) and a modulation laser pulse (wavelength and power) is also discussed. We demonstrate that an isolated TW-level as a hard X-ray can be produced by a properly chosen single current spike in an electron bunch with simulation results. By using realistic specifications of an electron bunch of the Pohang Accelerator Laboratory X-ray Free-Electron Laser (PAL-XFEL), we show that an isolated, >1.0 TW and ~36 as X-ray pulse at 12.4 keV can be generated in an optimized-tapered undulator line. This result opens a new vista for current XFEL operation: the attosecond XFEL.

## Introduction

X-ray free-electron laser (XFELs) facilities^1–6^ currently supply intense femtosecond XFEL pulses with a few tens of gigawatt power, generated in the self-amplified spontaneous emission (SASE) process^7^. Even though XFELs are excellent sources, allowing the study of new phenomena in the fields of physics, chemistry, biology and material science, these pulses are still too slow to follow the electron dynamics in atoms, molecules, and nanoscopic systems in real time. To follow electronic motion, an attosecond X-ray pulse is requisite. For example, Fratalocchi and Ruocco^8^ recently showed that single-molecule imaging to determine the structure of molecules is only possible with an attosecond hard X-ray pulse. One of the immediate applications of such a pulse is the observation of real-time changes in the probability distribution of an electron’s position^9,10^. This will constitute a great advance in time-resolved diffraction experiments, enabling four-dimensional imaging with picometer spatial and attosecond temporal resolutions^9,11^. Such attosecond X-ray pulses would allow investigation of phenomena in ultrafast science and X-ray nonlinear science that have not yet been explored.

There have been several proposals to generate attosecond XFEL (as XFEL) pulses^12–24^, and the generation of single spike pulses has been demonstrated experimentally^25,26^. Most of these proposals aimed to produce XFEL radiation with powers at the gigawatt level or durations longer than 100 as. The number of photons per pulse in these cases is on the order of 10^8^. For a sufficient number of photons to be delivered to samples, the power of an as XFEL pulse should be on the order of terawatt (TW)^27,28^. Moreover, scientists desire to use an isolated as XFEL with a pulse duration of less than 100 as in order to observe ultrafast dynamical changes in the atomic scale world^8,29,30^.

Tanaka^31^ suggested a method to generate an isolated TW as XFEL pulse. This scheme includes a slotted foil and E-SASE^32^ section to generate many current spikes, and optical and electron beam delay units to delay X-ray pulses and electron beam. However, this method inherently generates a train of X-ray pulses, but cannot supply an isolated X-ray pulse. Furthermore, in reality, one may face numerous difficulties in the operation of optical delay units and magnet chicanes, due to the stability and control accuracy of X-ray mirror mounts and the power supply for the magnets. Prat *et al*.^33,34^ proposed two simpler schemes to generate TW as XFEL pulses. They used an irregularly spaced slotted foil to deteriorate the emittance in several parts of an electron beam. However, they did not employ a laser pulse to generate an electron current spike. Thus, these two methods inherently pose challenges in synchronization between an X-ray pulse and an optical pump laser. Such a synchronization is of fundamental importance in pump-probe experiments. Recently, Tanaka, Prat and Parc *et al*.^35^ suggested a different idea for an as XFEL pulse, combining the two ideas proposed in refs^31,33^. Three methods are proposed to make irregularly spaced current peaks. One of the methods that uses a chirped laser pulse has also been published independently^36^.

Kumar *et al*.^37^ proposed a simpler method for a TW as XFEL with a single current spike, yet still utilizing delay units for electrons and photons. They use a high peak current spike, such as 30 kA, generated in the enhanced-SASE (E-SASE) section^32^.

Although all of these proposals constitute innovative approaches, their realization may face difficulties because their systems are complex, including optical mirror mounts, chicanes, power supplies which will decrease the accuracy in the control of the electron spikes or X-ray pulse down to few tens of nm. It is then natural to pose the following question: without any delay unit, can a single current spike produce TW as XFEL pulse? The following open questions need to be addressed: (1) how short should the current spike be? (2) how much high peak current is needed and how can it be made? (3) how high does the power of a modulation laser need to be? (4) what is the best wavelength for the modulation laser?

**Figure 1:**
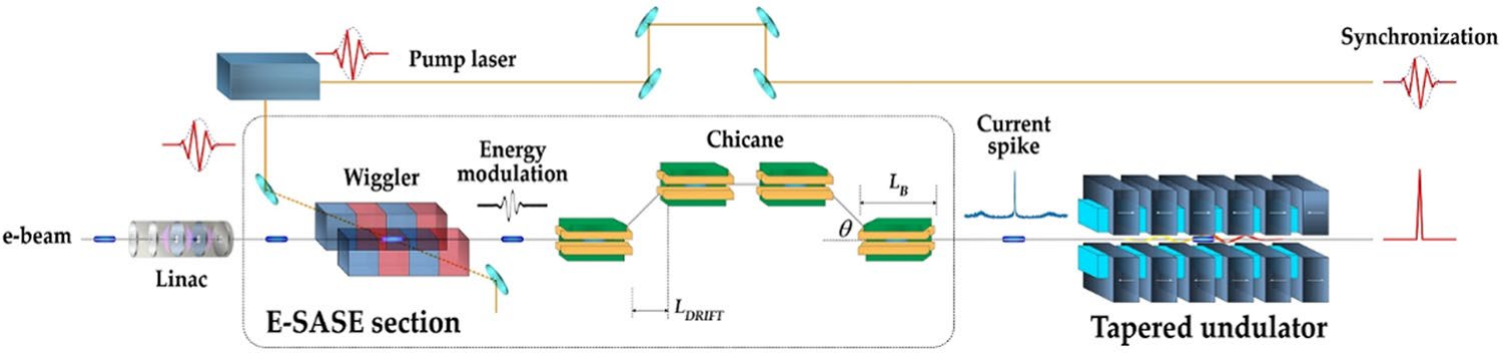
Schematic layout for the generation of synchronized TW as XFEL with E-SASE section and undulator tapering.

In this paper, we address all of these questions and propose the simplest scheme for the generation of an isolated TW as XFEL pulse without any optical or electron delay unit between undulator modules, as shown in Fig. 1. The most important feature in the illustration is that there are no X-ray mirrors (optical delay units) or magnetic chicane (electron delay unit) in an undulator line. Because there is no delay unit, no stability issues associated with a delay unit will arise in the real operation of XFEL. This design constitutes a realistic solution to realize a TW as XFEL. This design provides another benefit in the XFEL operation mode: flexibility in the change of XFEL operation mode from normal SASE mode to as XFEL mode.

This scheme will open a new regime of XFEL operation: TW as XFEL.

## Results

### Current spike issues

**Figure 2:**
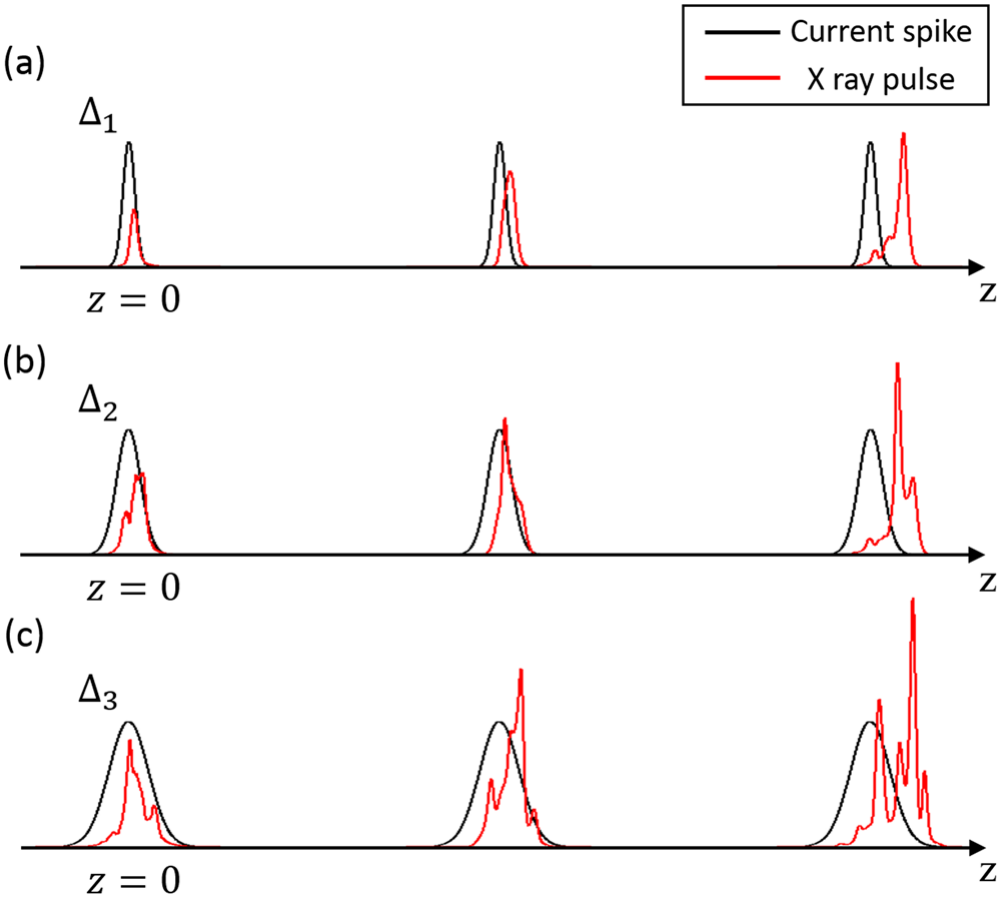
Schematic illustration of the power growth of an X-ray pulse under a single current spike: Δ_1_ < Δ_2_ < Δ_3_. The wider is the current spike width, the higher is the X-ray pulse power, at the cost of a poor temporal structure and a longer pulse duration.

We set the target values of a TW as X-ray pulse as follows: the power is larger than 1 TW, and the pulse duration is shorter than 100 as. Our scheme is based on the weak superradiant regime of operation^38,39^. To realize these difficult target values in the regime, we investigate a criterion for a current spike. Optimal parameters should be established for the width and height of a current spike. It is helpful to examine how an X-ray pulse develops under a single current spike. Schematic views are presented in Fig. 2.

For a current spike with small Δ_1_ (narrow width), as shown in Fig. 2(a), the width of an X-ray pulse would be small; however, the power would also be limited and low. With a narrow width of a current spike, the X-ray pulse does not have a sufficient opportunity to be amplified along the undulator line because the X-ray pulse slips away exactly one wavelength per one undulator period due to the FEL resonance condition^40^. After several undulator periods, the X-ray pulse slips away completely from the current spike. Figure 2(b) shows that a current spike with a larger width Δ_2_ than Δ_1_ would make a wider X-ray pulse than that in Fig. 2(a). The power of the X-ray pulse would be also greater than that in Fig. 2(a). The origin of this difference in radiation power between the two cases constitutes the duration time of the X-ray pulse in the current spike.

It could reasonably be concluded that the wider is the current spike width, the higher is the X-ray pulse power. However, it is necessary to consider another important aspect of the pulse: temporal shape. We aim to generate a single attosecond pulse with a pulse duration of less than 100 as. As shown in Fig. 2(c), if the width of a current spike is too large, extra radiation spikes are developed besides a single main spike, resulting in a long pulse with a poor temporal structure. This consideration indicates that there would be an optimal value for the width of a current spike.

To elucidate how the power and the width of an XFEL pulse changes with respect to the change of the width and the height of the current spike, a series of FEL simulations have been carried out for different widths and heights of an ideal Gaussian current spike. For FEL simulations, GENESIS^41^, which is a 3D time-dependent FEL simulation code, is used. We first need to determine the appropriate width (**Δ**) of a current spike. Several Gaussian current spikes with different widths for a fixed peak current (35 kA, in this case) are utilized in the simulation, adopting PAL-XFEL parameters (Table S1 in Supplementary Information).

**Figure 3:**
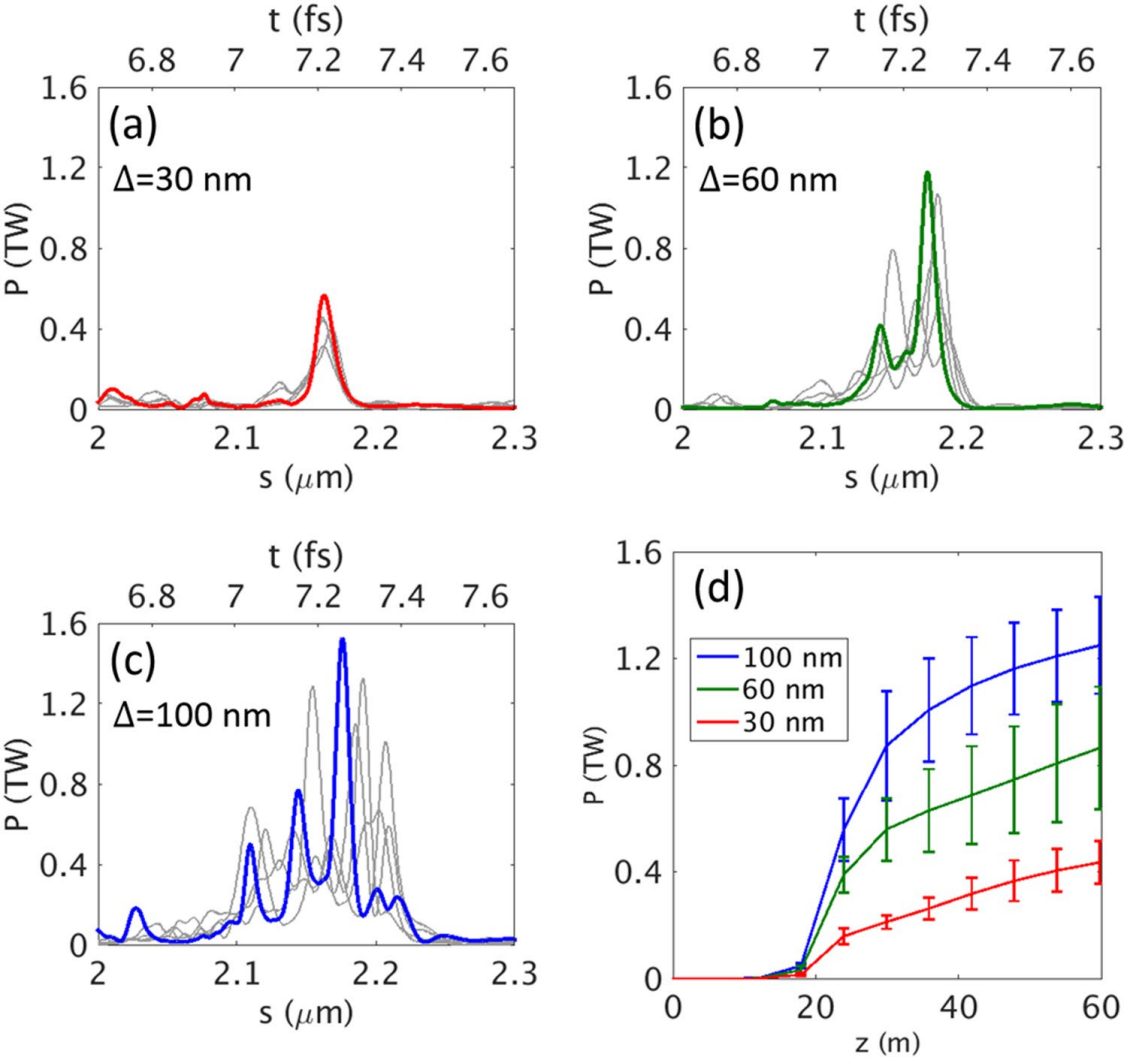
The shape of the radiation pulse and fluctuations at the end of the undulator line. Temporal structure (in bright color) and fluctuations (in grey color) of a radiation pulse at the end of the undulator line are shown for different current widths (**a**) 30 nm, (**b**) 60 nm, and (**c**) 100 nm. (**d**) The average peak radiation power for different current widths at a current of 35 kA. The error bars come from the results with five different random seeds.

The results are summarized in Fig. 3. As shown in Fig. 3(a), for a current width of Δ = 30 nm, the width of the X-ray pulse is ~50 as, which is much less than 100 as, with a temporal shape of a single spike; however, the FEL power reaches only ~0.56 TW. For a current width of Δ = 60 nm (Fig. 3(b)), the radiation power reaches more than ~1.2 TW, and the width is still less than 100 as. However, for a current width of Δ = 100 nm (Fig. 3(c)), the temporal structure is poor and the pulse duration is long, ~300 as. As discussed above, this indicates that an optimum width exists for a current spike, which constitutes a trade-off between the peak power and the temporal structure. The growth of the peak power of the X-ray pulse is presented in Fig. 3(d) for different current widths. We note that the peak XFEL power increases as the current spike width becomes wider. To keep the XFEL width within 100 as, the above analysis suggests that the width should be between 30 nm and 100 nm. Here, we choose Δ = 60 nm.

To obtain high radiation power within short slippage length, FEL parameter *ρ*^42^ has to be increased to shorten the gain length and enhance the saturation power^43^ (details are described in Supplementary Information). By using E-SASE section, this can be accomplished by increasing the height of the current spike. With respect to the issue related to the height of the current spike, several Gaussian current spikes with different peak current values for a fixed width (60 nm) have been examined in a series of FEL simulations by using GENESIS^41^ code. As expected, peak power increases as peak current becomes higher (data not shown). When the peak current is higher than 35 kA, radiation power becomes larger than 1 TW. Such a high peak current may suffer from the beam space-charge effect. However, ref.^44^ reveals that the effect can be negligible at a high beam energy such as 10 GeV in the present study.

### Modulation laser issues

**Figure 4:**
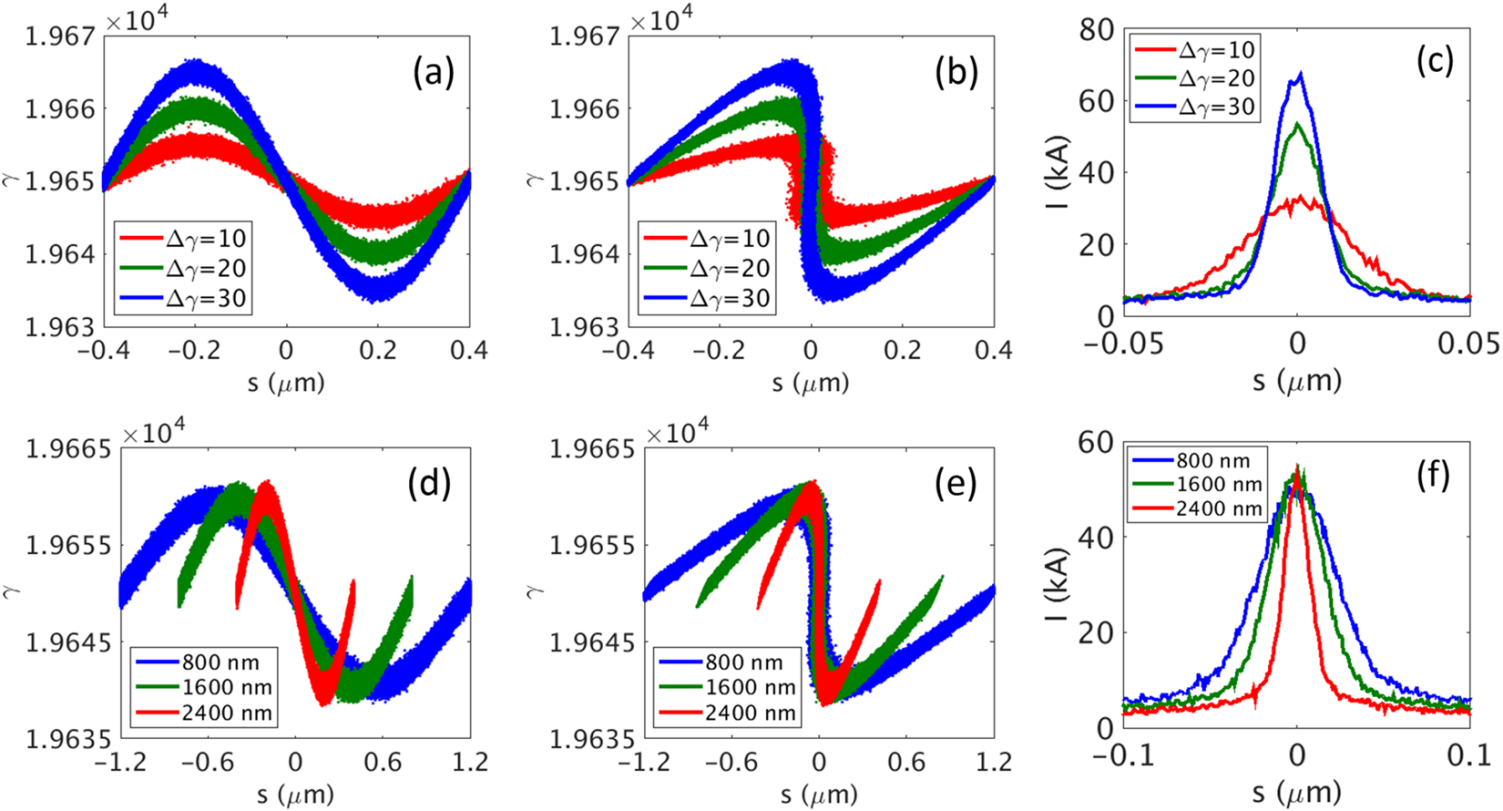
Effect of the energy and the wavelength of a modulation laser on the current spike formation. (**a**–**c**) Higher laser energy leads to higher energy modulation in the electron beam. Here, we examine the effect of the energy of a modulation laser in view of different values of energy modulation (Δγ) in the electron beam: Δγ = 10 (in red color), 20 (in green color), and 30 (in blue color) at a wavelength of 800 nm for the modulation laser. (**d**–**f**) Effect of the wavelength of the modulation laser: 800 nm (in red color), 1600 nm (in green color), and 2400 nm (in blue color) for Δγ = 20. (**a**,**d**) Energy of the electron beam is modulated after a wiggler. (**b**,**e**) The distribution is distorted in the phase space after a chicane. (**c**,**f**) Current profile of the electron beam after chicane, showing a current spike.

We now discuss the laser parameters to realize the single current spike with a width of Δ = 60 nm and a peak current of 35 kA. In the wiggler of the E-SASE section, the electron beam interacts with a few-cycle laser, whose wavelength is the same as the resonance condition of the wiggler. The electron beam energy increases or decreases according to the laser, and then the energy modulation along the electron beam is induced in the wiggler. In the chicane, such energy modulation converts to density modulation, and thus the electron bunch is reshaped in a chicane. Therefore, the properties of the wiggler and chicane must first be understood in relation to the change of electron beam parameters (see ‘Generation of current spike’ in Methods). A current spike formation occurring in a wiggler and a chicane is explained in Fig. 4 for different wavelengths of a modulating laser and different electron beam energy modulations (Δγ).

The effect of the energy of a modulation laser on the current spike formation is shown in Fig. 4(a–c). Figure 4(a,b) present the distribution of the electron beam after the wiggler and the chicane in the electron’s kinetic energy domain, respectively, for different energy modulations (Δγ = 10, 20, and 30) with a fixed laser wavelength of 800 nm. Figure 4(c) is the current profile in the electron density domain. The higher is the energy modulation for a given laser wavelength, the narrower is the current spike and the higher is the peak current^45^. Therefore, we can make a narrow current spike simultaneously with a higher peak current via a higher energy modulation, which is controlled by a modulation laser energy for a given wavelength.

The effect of a laser wavelength on the formation of a current spike for a given energy modulation is also investigated. The simulation results for different wavelengths (800, 1600, and 2400 nm) for a given energy modulation of Δγ = 20 are shown in Fig. 4(d–f). When a wavelength becomes longer, the width of a resultant current spike becomes wider. Note that the peak currents (heights in Fig. 4(f)) are almost the same for different wavelengths. Therefore, this reveals that a short wavelength is more suitable for a narrow current width, leading to a shorter XFEL pulse.

With the basic understanding about the formation of a current spike discussed above in relation to Fig. 4, we can propose two steps to realize a current spike with a width of 60 nm and a peak current of over 35 kA as follows.

First, we select a wavelength for the modulation laser, such as 800 nm, as shown in Fig. 4(a–c). We observe the increase of current with the increase of the laser energy, as presented in Fig. 4(c), and determine the energy modulation Δγ to obtain the peak current over 35 kA. A larger laser energy is needed for a larger Δγ. From Fig. 4(c), we note that Δγ = 20 is needed to obtain the current spike over 35 kA. However, the width of the current spike is only approximately 20 nm, which is too narrow for the sufficient amplification of radiation power to a TW. As the second step, we need to increase the current width by increasing the wavelength of the modulation laser, as shown in Fig. 4(f). However, it is necessary to maintain the energy modulation Δγ = 20 when we change the laser wavelength.

**Figure 5:**
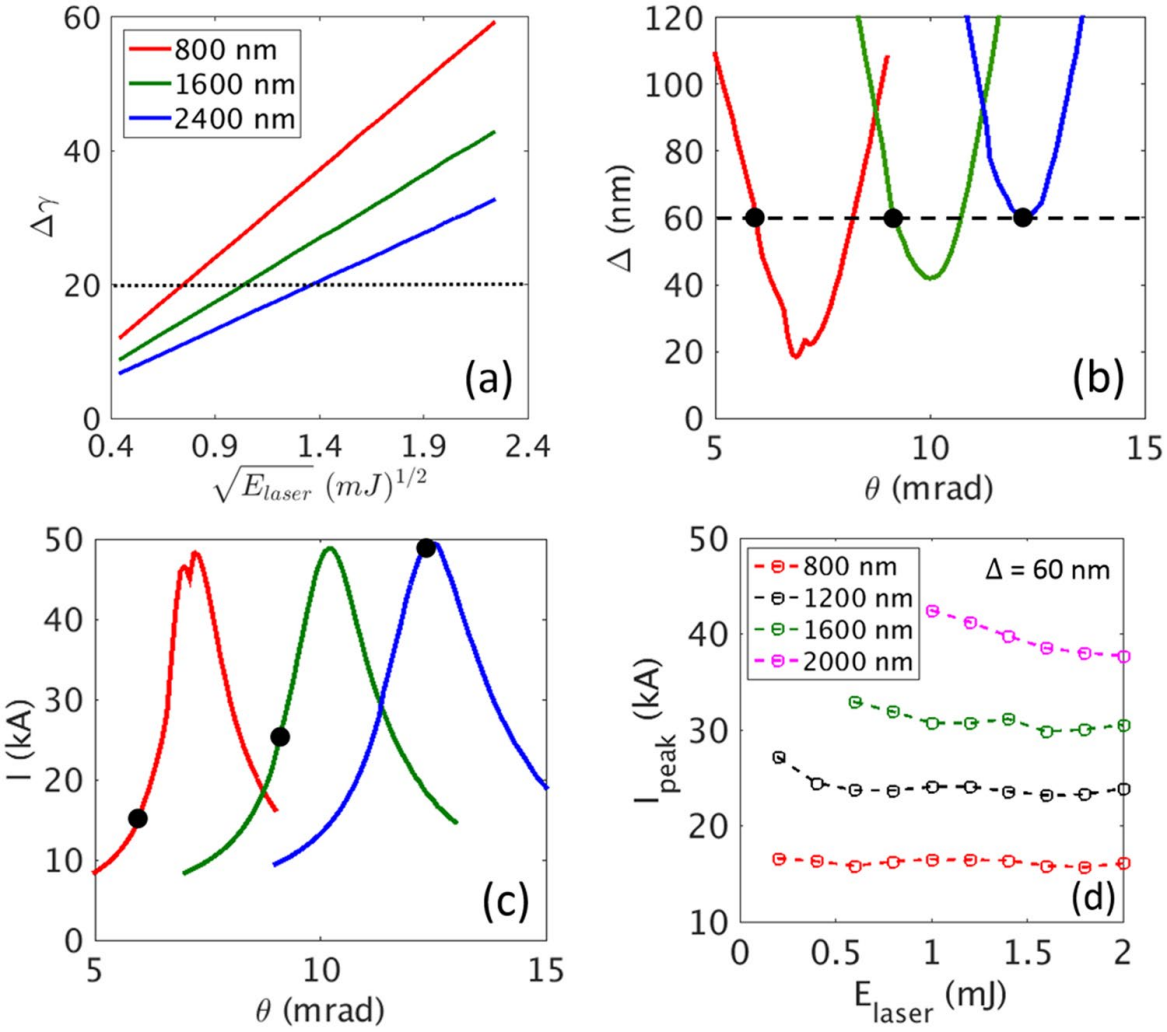
(**a**) Change of energy modulation after a wiggler with respect to the change of laser energy for different wavelengths. The black dotted line denotes the energy modulation Δγ = 20. (**b**) The width and (**c**) the peak current of the current spike with respect to the change of a bending angle in the chicane (see ‘Generation of current spike’ in Methods). The values of Δγ are 20 for (**b**) and (**c**). The black dashed line is the current width Δ = 60 nm. The black filled dots in (**b**) denote the cases of different wavelengths with a current spike width of 60 nm. The black filled dots in (**c**) denote the same cases as in (**b**). (**d**) The peak current that we can obtain for several values of laser energy for different wavelengths for a given current spike width (Δ = 60 nm). The circles in (**d**) are determined by the same procedure as in (**b**) and (**c**).

The relation between the energy modulation and the laser energy is shown in Fig. 5(a) for different wavelengths. The laser energy has to be increased as the laser wavelength increases to obtain the same Δγ. How the width and the height of a current spike changes with respect to the bending angle (*θ* in Fig. 1) is shown in Fig. 5(b,c) for the different wavelengths for a given energy modulation of Δγ = 20. For example, for the case of λ = 1600 nm, the minimum width of Δ = 42 nm is obtained at a bending angle of ~10 mrad with a peak current of ~50 kA. The minimum width for a longer wavelength is larger (Fig. 5(b)), while the maximum of the peak current is similar (Fig. 5(c)).

It is interesting to note from Fig. 5(a–c) that a current width of Δ = 60 nm can be achieved for different laser wavelengths, as shown in Fig. 5(b). However, in the case of a shorter wavelength (for example, 800 nm), for a current spike width of Δ = 60 nm, the bending angle (*θ*) has to be set at ~6.3 mrad and the corresponding peak current is 15 kA. Although a higher laser energy is needed to make the same amount of energy modulation for a longer wavelength, as shown in Fig. 5(a), a longer laser wavelength is actually preferred to obtain a high peak current for a given current width. For a laser energy of ~2.2 mJ of 2400 nm light, we are able to obtain Δγ = 20, a current spike of Δ = 60 nm, and a peak current of ~50 kA at a bending angle of ~12 mrad.

To understand the dependence of the peak current on laser energy for different wavelengths, a series of simulations for the wiggler and chicane are performed by using ELEGANT code^46^, as shown in Fig. 5(d), when the current width is fixed at 60 nm. Five different wavelengths (800, 1200, 1600, 2000, and 2400 nm) are considered in this study. If we fix the width of the current spike for a given laser wavelength, we obtain similar values of peak current, regardless of laser energies by adjusting the bending angle. This is surprising because it implies that a small laser energy can be utilized to make a higher current for a given width of a current spike.

We also note that to obtain a given width at a given wavelength, there is a lower limit in the laser energy, below which such a width cannot be achieved. The limit increases as a wavelength increases. For example, no data are given for an energy below 0.6 mJ in the case of 1600 nm: we cannot obtain a current width of 60 nm with a laser energy less than ~0.6 mJ. For a wavelength of 2400 nm, which is not shown, a current spike with a width of 60 nm cannot be generated even with a laser energy of 2 mJ. This means that we need a laser energy larger than 2 mJ.

To make a current spike with a width of 60 nm and energy modulation Δγ = 20, a laser with a wavelength shorter than 2400 nm will suffice, as shown in Fig. 5(b). To make a higher peak current than 35 kA, it is necessary to use a laser with a wavelength longer than 1600 nm, as shown in Fig. 5(d). We choose a 2000 nm wavelength in this study. In summary, we can make any arbitrary current spike by adjusting the wavelength and the energy of a laser, and the bending angle of a chicane.

### Radiation simulation

**Figure 6:**
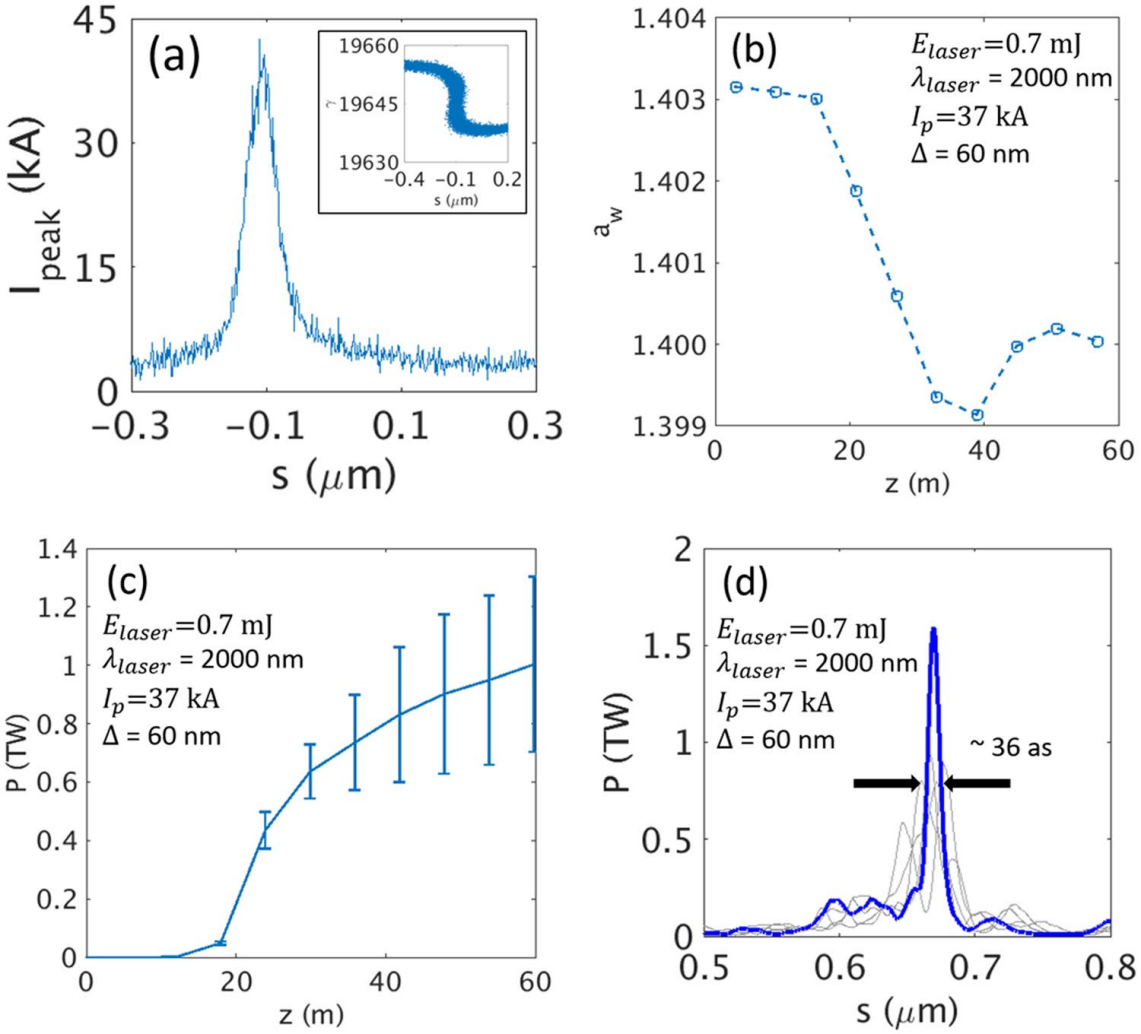
FEL simulation results in the PAL-XFEL configuration. (**a**) Optimized current spike profile of the electron beam with 2 um, 0.7 mJ few-cycle laser. Phase space distribution corresponding to the current spike is plotted in the inset. (**b**) Undulator parameter of optimized tapering. (**c**) Peak radiation power averaged over five simulations with error bars along the undulator length. (**d**) Representative shape (blue) and fluctuation (grey) of a radiation pulse at the end of the undulator line.

According to the steps which are proposed in the previous section, the optimized current spike for TW as XFEL (>35 kA, ~60 nm) can be obtained in the PAL-XFEL configuration with a laser wavelength of 2 um and a laser energy of 0.7 mJ. Bending angle *θ* of the chicane in E-SASE section is 0.72°. The current spike profile and the phase space distribution of the electron beam are presented in Fig. 6(a). Because there is only one single current spike and there is no X-ray mirror in the undulator line, an optimized tapering in an undulator line is required to maximize the radiation power. The optimization of an undulator tapering (see ‘Tapering optimization’ in Methods) is shown in Fig. 6(b). The shape of the optimized tapering function is not quadratic. After z = 40 m, the undulator parameter is increased again. With optimized tapering, the peak power of XFEL radiation and its temporal structure are presented in Fig. 6(c,d), respectively. The averaged peak XFEL power reaches up to 1.0 ± 0.3 TW, as shown in Fig. 6(c). The temporal structure of the X-ray pulses exhibits a single spike of ~36 as pulse duration (FWHM), as presented in Fig. 6(d). These results are impressive because we did not use an optical mirror or chicane magnet in the undulator line, as shown in Fig. 1.

## Discussion

We investigated the possibility that an isolated TW as XFEL hard X-ray pulse can be generated by a single current spike without the assistance of an optical delay unit and chicane. This investigation reveals the conditions of the current spike and the modulation laser: the peak current should be higher than 35 kA and the current spike width should be fixed as 60 nm. If the current spike width is shorter than 60 nm, the final XFEL power cannot reach 1 TW. If the current spike width is wider than 60 nm, the XFEL radiation has several spikes and the length would be over 100 as.

High peak current in the chicane emits coherent synchrotron radiation (CSR), and this may constitute a problem in FEL performance. However, CSR from such high peak current has no noticeable effects on the electron beam^47^ and can be manageable by coupling of transverse and longitudinal dynamics in the chicane^48^ or reducing the length of the dipole magnet of the chicane^49^. Therefore, CSR does not seriously affect our method, and it accords with the simulation results from ELEGANT code, which includes the CSR effect, as shown in Fig. 6(a).

For the PAL-XFEL case, when the wavelength of a laser is 2000 nm and the laser energy is 0.7 mJ, we can obtain a current spike whose peak value is 37 kA and width is 60 nm. In the process of energy modulation in the wiggler, electrons in one phase of the laser cycle gain a certain amount of energy; however, about the same amount of energy is taken away from electrons in the opposite phase of the laser cycle. Thus it is likely that the intensity of the laser is more or less constant during the interaction with electron beam. Indeed, we demonstrated that a single XFEL of 36 as (FWHM) pulse width over 1.0 TW peak power can be generated with an E-SASE scheme and the assistance of optimized tapering.

The proposed scheme in this study is simple and efficient, and can be easily implemented in existing XFELs. Moreover, because there is no change in an undulator line, the normal operation of the XFEL is not disturbed by the upgrade from the normal SASE XFEL to TW as XFEL. These results will substantially increase the flexibility of the operation of XFEL, enabling a new regime of XFEL operation: the attosecond XFEL. This will broaden the scope of ultrafast science and offer new possibilities for single molecule imaging.

## Methods

### Generation of current spike

An electron beam (‘e-beam’ in Fig. 1) is accelerated up to GeV level (10 GeV for PAL-XFEL) from a linear accelerator. A few-cycle laser pulse is transported to a wiggler to make an energy modulation in the electron beam. The laser wavelength is the same as the resonance condition of the wiggler. The energy distribution of the electron beam along the beam direction is modulated from the interaction of the electric field of a few-cycle laser pulse with the electrons in the wiggler. The synchronization between a pump laser and an XFEL is inherently realized since the pump pulse and the modulation pulse are generated from the same laser^37,50,51^.

The energy modulation in the wiggler is converted to the charge density modulation in ‘Chicane’ in Fig. 1. The electrons with different kinetic energies follow different paths in the four dipole magnets. This path difference constitutes the origin of the charge density modulation. The relative difference in the electron paths *Δs* is calculated as follows:

1 $${\rm{\Delta }}s={R}_{56}\frac{{\rm{\Delta }}E}{E}$$

where *E* is the average energy of the electron beam; and *ΔE* is the difference from the average energy of the electron beam. *R*_56_ is the longitudinal dispersion, and is defined as^52^:

2 $${R}_{56}=2{\theta }^{2}({L}_{DRIFT}+\frac{2}{3}{L}_{B})$$

where *θ* is the bending angle of the chicane; *L*_*DRIFT*_ is the drift length between the first and the second (or the third and the fourth) bending magnet in the chicane; and *L*_*B*_ is the length of the bending magnet. By using equations (1) and (2), we discuss the optimal values for a wavelength and pulse energy of a modulation laser.

**Figure 7:**
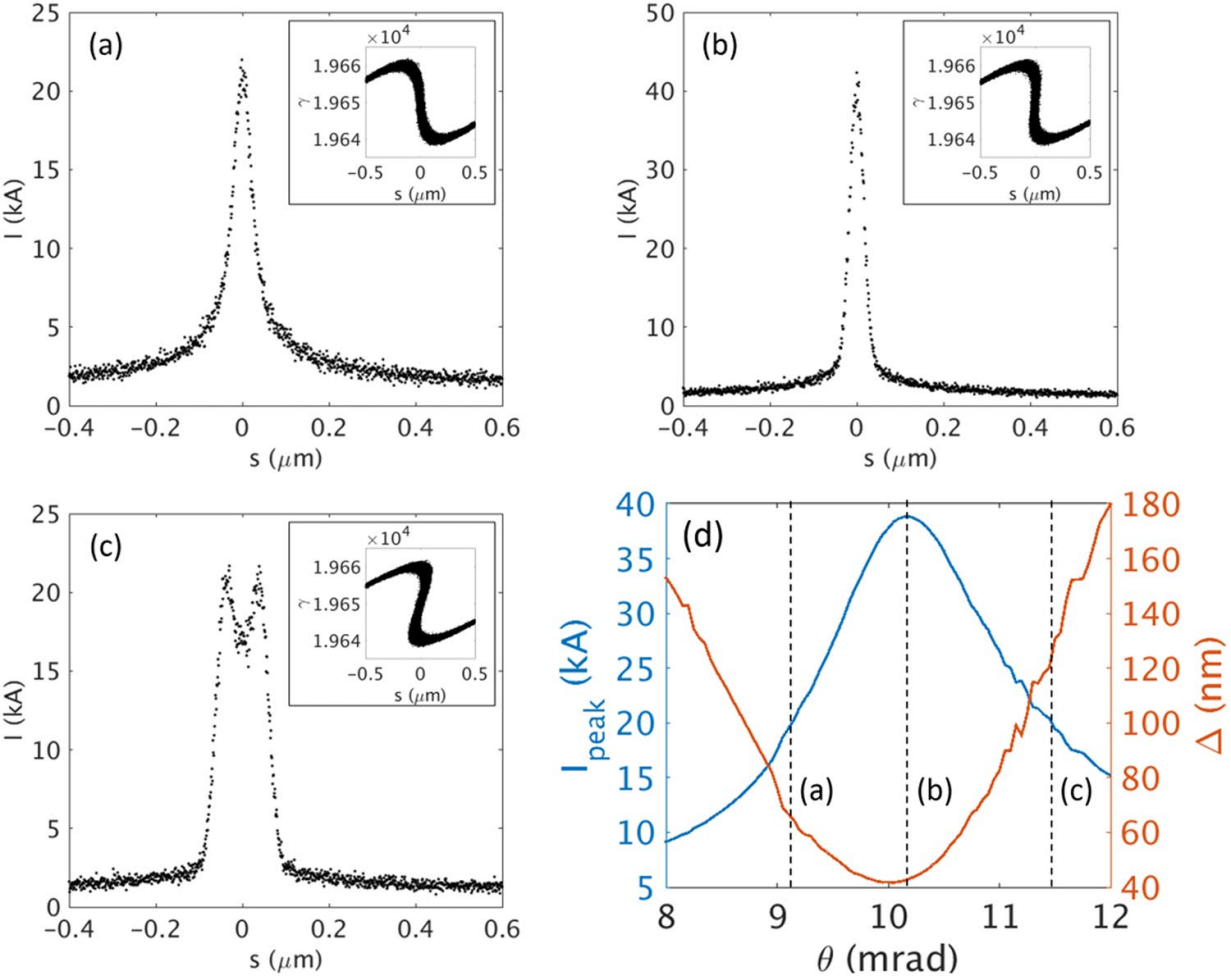
Shape of current spike for (**a**) weak bunching, (**b**) maximum bunching, and (**c**) over-bunching. Phase space of each case is plotted in the inset. (**d**) Peak current (blue line) and width (red line) of a current spike with respect to the bending angle of the chicane. The black dashed lines indicate the cases shown in (**a**),(**b**) and (**c**).

The generation of a current spike has been simulated, and the results are presented in Fig. 7. The PAL-XFEL parameters (Table S1 in Supplementary Information) are used in the simulation. When the e-beam is passing through the chicane, the e-beam is bunched, as shown in Fig. 7(a). When we increase the bending angle, the peak current is increased further, as shown in Fig. 7(b). However, if we increase the bending angle more, the e-beam is over-bunched, and the peak current becomes smaller again. In addition, the e-beam exhibits two peaks in the top part, as shown in Fig. 7(c). The change of the width and the peak current of the current spike with respect to the bending angle is plotted in Fig. 7(d). Note that for the over-bunched case (Fig. 7(c)), there is an ambiguity to determine the pulse width from the Gaussian fitting because of the two peaks on the top of the bunched area. This is the origin of the ragged curve for the high bending angle. By controlling the bending angle, we can adjust the width and peak current of the current spike.

### Tapering optimization

The objective of undulator tapering is to maintain the resonance condition between an electron beam and its X-ray radiation for the effective power growth of the X-ray radiation^53–55^. When the electron beam has the same current over the whole electron beam, the electron beam energy is lost uniformly over the entire electron beam due to X-ray radiation. Therefore, a quadratic undulator tapering is usually applied to compensate for such an energy loss. In contrast, when the E-SASE is utilized, the peak current along the electron beam is not uniform and changes drastically from one place to another due to current spikes. In this case, a quadratic tapering does not work, but rather a careful optimization of tapering is requisite.

The optimization process of each undulator module is as follows. We first measure the electron beam energy of the current spike at the entrance of an undulator (γ_ent_) and calculate the initial rms undulator parameter (*a*_w,init_), which is given by:

3 $${{\rm{a}}}_{{\rm{w}},{\rm{init}}}=\sqrt{\frac{2{{\rm{\gamma }}}_{{\rm{ent}}}^{2}{{\rm{\lambda }}}_{{\rm{r}}}}{{{\rm{\lambda }}}_{{\rm{u}}}}-1}$$

where *λ*_r_ is the wavelength of the X-ray; and *λ*_*u*_ is the undulator period. The second step is to measure the electron beam energy of the current spike at the end of the undulator (γ_end_) and calculate the undulator parameter (*a*_w,cal_) by using the average value of the electron beam energy in the undulator (γ_ave_ = (*γ*_ent_ + *γ*_end_)/2) as follows:

4 $${{\rm{a}}}_{{\rm{w}},{\rm{cal}}}=\sqrt{\frac{2{{\rm{\gamma }}}_{{\rm{ave}}}^{2}{{\rm{\lambda }}}_{{\rm{r}}}}{{{\rm{\lambda }}}_{{\rm{u}}}}-1}$$

If *a*_w,cal_ and *a*_w,init_ have the same value, this means that the resonance condition in the current spike is well satisfied, and we can fix the rms undulator parameter as *a*_w,cal_. If not, we have to replace *a*_w,init_ with *a*_w,cal_, and repeat the second step until *a*_w,cal_ and *a*_w,init_ have the same value.

**Figure 8:**
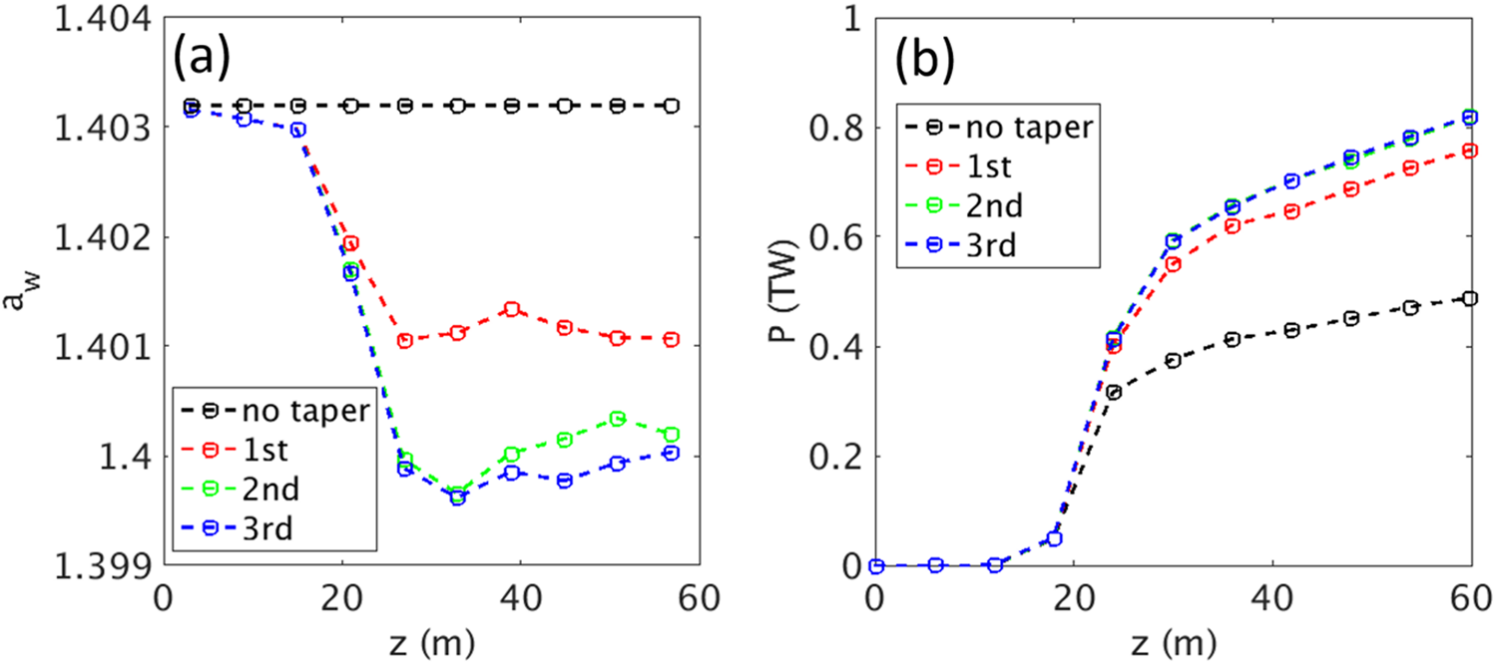
Undulator tapering optimization process (**a**) undulator parameter and (**b**) radiation power. Three iterations have already led to good convergence both in X-ray radiation power and undulator parameters.

In the simulation, the optimization process simultaneously proceeds for all of the undulator modules. After three iterations, the undulator parameter and the radiation power are converged because the resonance condition in each undulator module is well matched, as shown in Fig. 8. In the real experiment, the electron beam energy of the current spike can be measured by using a transverse deflecting structure and dispersion section, such as a bending magnet^56^. With these elements, the longitudinal phase space of the electron beam can be reconstructed, and the electron beam energy of the current spike is also determined experimentally. Then, this tapering method is easily realized when the optimization process is conducted one-by-one.

## Electronic supplementary material

Supplementary Information
