# Author Correction: Isolated terawatt attosecond hard X-ray pulse generated from single current spike

**DOI:** 10.1038/s41598-019-44364-3

**Published:** 2019-06-06

**Authors:** Chi Hyun Shim, Yong Woon Parc, Sandeep Kumar, In Soo Ko, Dong Eon Kim

**Affiliations:** 1 Department of Physics, Pohang University of Science and Technology, Pohang, 37673 Korea; 2 Pohang Accelerator Laboratory, Pohang University of Science and Technology, Pohang, 37673 Korea; 3 Department of Physics, Ulsan National Institute of Science and Technology, Ulsan, 44919 Korea; 4 Department of Physics, Center for Attosecond Science and Technology, Pohang University of Science and Technology, Pohang, 37673 Korea; 5 Max Planck POSTECH/Korea Res. Init., Pohang, 37673 Korea

Correction to: *Scientific Reports* 10.1038/s41598-018-25778-x, published online 10 May 2018

This Article contains an error in Figure 4 where the key is incorrect in panels (d), (e), and (f). The correct Figure 4 appears below as Figure 1.

**Figure 1:**
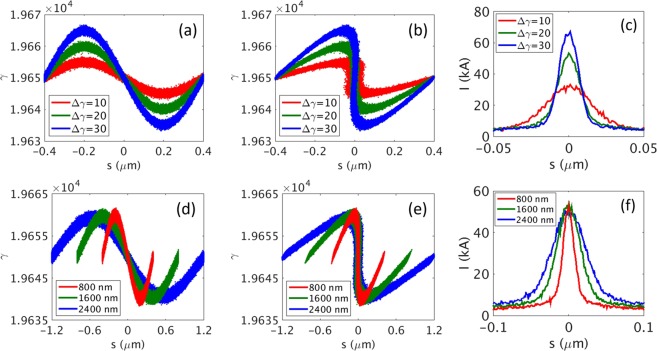
Effect of the energy and the wavelength of a modulation laser on the current spike formation. (**a**–**c**) Higher laser energy leads to higher energy modulation in the electron beam. Here, we examine the effect of the energy of a modulation laser in view of different values of energy modulation (Δγ) in the electron beam: Δγ = 10 (in red color), 20 (in green color), and 30 (in blue color) at a wavelength of 800 nm for the modulation laser. (**d**–**f**) Effect of the wavelength of the modulation laser: 800 nm (in red color), 1600 nm (in green color), and 2400 nm (in blue color) for Δγ = 20. (**a**,**d**) Energy of the electron beam is modulated after a wiggler. (**b**,**e**) The distribution is distorted in the phase space after a chicane. (**c**,**f**) Current profile of the electron beam after chicane, showing a current spike.

