# Supplementary material for: Isolated terawatt attosecond hard X-ray pulse generated from single current spike
Source: Sci Rep. 2018 May 10;8:7463. doi: 10.1038/s41598-018-25778-x (PMC5945633; doi:10.1038/s41598-018-25778-x)
Supplement: Supplementary file 1 — Supplementary Information [file 41598_2018_25778_MOESM1_ESM.pdf]

Supplementary Information for

Isolated terawatt attosecond hard X-ray pulse generated from single current spike

Chi Hyun Shim,[1] Yong Woon Parc,[2,*] Sandeep Kumar,[3] In Soo Ko,[1] and Dong Eon Kim[4,5,*]

[1]Department of Physics, Pohang University of Science and Technology, Pohang, 37673, Korea
[2]Pohang Accelerator Laboratory, Pohang University of Science and Technology, Pohang, 37673, Korea
[3]Department of Physics, Ulsan National Institute of Science and Technology, Ulsan, 44919, Korea
[4]Department of Physics, Center for Attosecond Science and Technology, Pohang University of Science and Technology, Pohang, 37673, Korea
[5]Max Planck POSTECH/Korea Res. Init., Pohang, 37673, Korea
*Corresponding author: young1@postech.ac.kr, kimd@postech.ac.kr

Pohang Accelerator Laboratory X-ray Free-Electron Laser (PAL-XFEL) is currently operating facility which has two undulator lines: hard X-ray undulator line with 10 GeV electron beam and soft X-ray undulator line with 3.15 GeV electron beam. In our research, PAL-XFEL parameters for hard X-ray undulator line are used in simulation and listed in Table S1.

Table S1. Parameters for PAL-XFEL used in simulations.

**Parameter**	**Value**		**Unit**
	SASE	E-SASE for current spike	
Electron beam			
Energy	10		GeV
Charge	200		pC
Peak current	3	35	kA
rms normalized slice emittance ($\varepsilon_{x,y}$)	0.25		μm
rms slice energy spread	250	2917	keV
Wiggler			
Period	0.5		m
Length	1.0		m
Chicane			
Bending magnet length (L_B in Fig. 1)	0.3		m
Drift length (L_{DRIFT} in Fig. 1)	2.5		m
Undulator			
Period	26		mm
a_w	1.403		
Length	4.94		m
Radiation			
Wavelength (photon energy)	0.1 (12.4)		nm (keV)
FEL parameter (ρ)	6.2×10^{-4}	1.4×10^{-3}	
Gain length (L_g)	2.18	0.94	m
Saturation length (L_{sat})	40.39	19.03	m
Saturation power (P_{sat})	23.18	639.1	GW

FEL parameter ρ is defined as follows[42]:

$$\rho = \left[\left(\frac{I}{I_A}\right)\left(\frac{\lambda_w A_w}{2\pi\sigma_x}\right)^2\left(\frac{1}{2\gamma_0}\right)^3\right]^{1/3} \tag{S1}$$

where I is the peak current, $I_A = 17.045$ kA is Alfven current, and λ_w is the undulator period. For the helical undulator, A_w has same value as rms undulator parameter, a_w. For the planar undulator, A_w has the value of $a_w[J_0(\xi) - J_1(\xi)]$ by using Bessel function where $\xi = a_w^2/2(1 + a_w^2)$. σ_x is the transverse electron beam size and the value is calculated by beta function and emittance as follows: $\sigma_x = \sqrt{\beta\epsilon}$. γ_0 is Lorentz factor of the electron beam. 1-dimensional gain length L_{1d} can be estimated by FEL parameter ρ as shown in Eq. (S2).

$$L_{1d} = \lambda_w/4\pi\sqrt{3}\rho \tag{S2}$$

By considering multidimensional effect, 3-dimensional gain length L_g can be estimated. There are scaling function between 1-dimensional FEL gain length and 3-dimensional FEL gain length as shown in Eq. (S3).

$$\frac{L_{1d}}{L_g} = F(\eta_d, \eta_\epsilon, \eta_\gamma) \tag{S3}$$

Such scaling function can be expressed as fitting formula based on the variational solution of the FEL dispersion relation by Ming Xie[43] as shown in Eq. (S4) with three parameters: Diffraction parameter $\eta_d = \frac{L_{1d}}{L_r}$, angular spread parameter $\eta_\epsilon = \left(\frac{L_{1d}}{\beta}\right)\left(\frac{4\pi\epsilon}{\lambda}\right)$, and energy spread parameter $\eta_\gamma = 4\pi\left(\frac{L_{1d}}{\lambda_w}\right)\left(\frac{\sigma_e}{E_0}\right)$.

$$\frac{L_{1d}}{L_g} = \frac{1}{1+\eta} \tag{S4}$$

The frequency detuning parameter is optimized to obtain the minimum FEL gain length. Rayleigh range in diffraction parameter is defined by using the resonant wavelength of FEL, λ: $L_r = 4\pi\sigma_x^2/\lambda$. β is beta function of the electron beam, ϵ is transverse emittance, σ_e is rms electron beam energy spread, and E_0 is electron beam energy. The gain length degradation factor η in Eq. (S4) is calculated by using fitting coefficients in Table S2 as shown in Eq. (S5).

$$\eta = a_1\eta_d^{a_2} + a_3\eta_\epsilon^{a_4} + a_5\eta_\gamma^{a_6} + a_7\eta_\epsilon^{a_8}\eta_\gamma^{a_9} + a_{10}\eta_d^{a_{11}}\eta_\gamma^{a_{12}} + a_{13}\eta_d^{a_{14}}\eta_\epsilon^{a_{15}} + a_{16}\eta_d^{a_{17}}\eta_\epsilon^{a_{18}}\eta_\gamma^{a_{19}} \tag{S5}$$

Table S2. Coefficient for Ming Xie's fitting formula of scaling function

$a_1 = 0.45$	$a_2 = 0.57$	$a_3 = 0.55$	$a_4 = 1.6$
$a_5 = 3$	$a_6 = 2$	$a_7 = 0.35$	$a_8 = 2.9$
$a_9 = 2.4$	$a_{10} = 51$	$a_{11} = 0.95$	$a_{12} = 3$
$a_{13} = 5.4$	$a_{14} = 0.7$	$a_{15} = 1.9$	$a_{16} = 1140$
$a_{17} = 2.2$	$a_{18} = 2.9$	$a_{19} = 3.2$	

Saturation power P_{sat} are also presented in Eq. (S6).

$$P_{sat} \approx 1.6\rho \left(\frac{L_{1d}}{L_g}\right)^2 P_{beam} \tag{S6}$$

P_{beam} is the power of the electron beam and is calculated by using the electron beam energy and peak current as follows: $P_{beam}[\mathrm{TW}] = E_0[\mathrm{GeV}]I[\mathrm{kA}]$

By increasing the peak current, FEL parameter value is increased as to 1.4×10^{-3} as shown in Table S1. Gain length is inverse proportional to FEL parameter as shown in Eq. (S2). Saturation power is proportional to $I^{4/3}$ because the power of the electron beam is also proportional to the peak current of the electron beam.

Multidimensional effect can be also estimated by using Ming Xie's fitting formula.[43] In our case, only electron beam energy spread is increased when the current spike is generated by E-SASE section. When the peak current is increased from 3 kA to 35 kA, electron beam energy spread is also increased from 250 keV to 2917 keV as shown in Table S1, Supplementary Information. From Eq. (S2), 1-dimensional FEL gain length is decreased from 1.92 m to 0.85 m. 3-dimensional FEL gain length, which is calculated by Ming Xie's fitting formula, is decreased from 2.18 m to 0.94 m with Eq. (S4). Although 3-dimensional FEL gain length is slightly longer than 1-dimensional FEL gain length when the peak current is 35 kA, it is still shorter than both of FEL gain lengths when the peak current is 3 kA. Therefore, higher peak current of current spike is still preferable to generate TW as XFEL in spite of multidimensional effect from electron beam energy spread.
